# Motivation of medical employees in the context of the COVID pandemic

**DOI:** 10.1192/j.eurpsy.2023.484

**Published:** 2023-07-19

**Authors:** S. Trifu, A. C. Trifu, A. Ciubară

**Affiliations:** ^1^Neurosciences; ^2^General Medicine, University of Medicine and Pharmacy Carol Davila, Bucharest; ^3^Psychiatry, Dunarea de Jos University of Medicine and Pharmacy, Galati, Romania

## Abstract

**Introduction:**

The Covid-19 pandemic has restructured the entire health care system. As systems of care were overwhelmed, many health professionals in related professional areas became increasingly involved in providing medical aid. We highlight a comparative analysis between two psychiatric wards of the same hospital, with qualified medical staff, with similar levels of competence, one of the wards caring for patients exclusively with major psychiatric pathologies, and the other patients with associated SARS-COV2 infection.

**Objectives:**

Considering that motivation at work depends on a multitude of factors, in our case, stress related to contamination, situational anxiety generated by psychiatric patients who arepartially inside on COVID infection, can decrease the motivation to perform a quality psychiatric medical act. Resilience and organizational civic behavior are instead variables with the role of maintaining professional motivation at an optimal level and enhancing dedication.

**Methods:**

We applied questionnaires aimed at motivation at work in relation to the dependent variables: stress, anxiety, resilience and civic organizational behavior in two different departmentsof the psychiatric hospital, a ward where patients were admitted positively confirmed COVID 19, versus a ward with non-COVID psychiatric patients.

**Results:**

Psychiatric medical staff are trained to treat predominantly psychomotor agitation, violent behavioral syndromes, suicide attempts, psychotic illnesses. 95% of the employees of theCOVID support department stated that they prefer to take care of 10 agitated patients, than one patient with COVID. The stress came in most cases from: insufficient supportequipment, insufficient doctor to coordinate nurses, lack of experience with somatic patients, fear of contamination generated by non-compliance with protective measures by psychiatric patients.

**Conclusions:**

Resilience and civic organizational behavior kept staff motivation at an optimal level, but lower than would have been appropriate. Also, motivation at work was lower compared tothe non-COVID psychiatric ward.

**Disclosure of Interest:**

None Declared

